# Influence of chronic lymphocytic thyroiditis on the risk of persistent and recurrent disease in patients with papillary thyroid carcinoma and elevated antithyroglobulin antibodies after initial therapy^☆^

**DOI:** 10.1016/j.bjorl.2017.05.005

**Published:** 2017-06-02

**Authors:** Marina Carvalho S. Côrtes, Pedro Weslley Rosario, Gabriela Franco Mourão, Maria Regina Calsolari

**Affiliations:** Santa Casa de Belo Horizonte, Serviço de Endocrinologia, Belo Horizonte, MG, Brazil

**Keywords:** Thyroid cancer, Chronic lymphocytic thyroiditis, Elevated antithyroglobulin, Persistent and recurrent disease, Câncer de tireoide, Tireoidite linfocítica crônica, Antitiroglobulina elevada, Doença persistente e recorrente

## Abstract

**Introduction:**

In patients with papillary thyroid carcinoma who have negative serum thyroglobulin after initial therapy, the risk of structural disease is higher among those with elevated antithyroglobulin antibodies compared to patients without antithyroglobulin antibodies. Other studies suggest that the presence of chronic lymphocytic thyroiditis is associated with a lower risk of persistence/recurrence of papillary thyroid carcinoma.

**Objective:**

This prospective study evaluated the influence of chronic lymphocytic thyroiditis on the risk of persistence and recurrence of papillary thyroid carcinoma in patients with negative thyroglobulin but elevated antithyroglobulin antibodies after initial therapy.

**Methods:**

This was a prospective study. Patients with clinical examination showing no anomalies, basal Tg < 1 ng/mL, and elevated antithyroglobulin antibodies 8–12 months after ablation were selected. The patients were divided into two groups: Group A, with chronic lymphocytic thyroiditis on histology; Group B, without histological chronic lymphocytic thyroiditis.

**Results:**

The time of follow-up ranged from 60 to 140 months. Persistent disease was detected in 3 patients of Group A (6.6%) and in 6 of Group B (8.8%) (*p* = 1.0). During follow-up, recurrences were diagnosed in 2 patients of Group A (4.7%) and in 5 of Group B (8%) (*p* = 0.7). Considering both persistent and recurrent disease, structural disease was detected in 5 patients of Group A (11.1%) and in 11 of Group B (16.1%) (*p* = 0.58). There was no case of death related to the disease.

**Conclusion:**

Our results do not support the hypothesis that chronic lymphocytic thyroiditis is associated with a lower risk of persistent or recurrent disease, at least in patients with persistently elevated antithyroglobulin antibodies after initial therapy for papillary thyroid carcinoma.

## Resumo

**Introdução:**

Em pacientes com carcinoma papilífero de tireoide e com tireoglobulina sérica negativa após a terapia inicial, o risco de doença estrutural é maior entre aqueles com anticorpos antitireoglobulina elevados em comparação com pacientes sem anticorpos antitireoglobulina. Outros estudos sugerem que a presença de tireoidite linfocítica crônica está associada a um menor risco de persistência/recorrência do carcinoma papilífero de teireoide.

**Objetivo:**

Este estudo prospectivo avaliou a influência da tireoidite linfocítica crônica sobre o risco de persistência e recorrência do carcinoma papilífero de tireoide em pacientes com tireoglobulina negativa, mas com anticorpos antitireoglobulinas elevados após a terapia inicial.

**Método:**

Esse foi um estudo prospectivo, no qual foram selecionados pacientes com exame clínico sem anomalias; tireoglobulina basal < 1 ng/mL e anticorpos antitireoglobulina elevados 8-12 meses após ablação. Os pacientes foram divididos em dois grupos: Grupo A, com tireoidite linfocítica crônica no exame histológico; Grupo B, histologicamente sem tireoidite linfocítica crônica.

**Resultados:**

O tempo de seguimento variou de 60 a 140 meses. Doença persistente foi detectada em 3 pacientes do Grupo A (6,6%) e em 6 do Grupo B (8,8%) (*p* = 1,0). Durante o seguimento, as recidivas foram diagnosticadas em 2 pacientes do Grupo A (4,7%) e em 5 do Grupo B (8%) (*p* = 0,7). Considerando tanto a doença persistente quanto a recorrente, doença estrutural foi detectada em 5 pacientes do Grupo A (11,1%) e em 11 do Grupo B (16,1%) (*p* = 0,58). Não houve nenhum caso de óbito relacionado à doença.

**Conclusão:**

Nossos resultados não apoiam a hipótese de que a tireoidite linfocítica crônica esteja associada a um menor risco de doença persistente ou recorrente, pelo menos em pacientes com anticorpos antitireoglobulina persistentemente elevados após a terapia inicial do carcinoma papilífero de tireoide.

## Introduction

In patients with papillary thyroid carcinoma (PTC) who have negative serum thyroglobulin (Tg) after initial therapy, the risk of structural disease is significantly higher among those with elevated antithyroglobulin antibodies (TgAb) compared to patients without TgAb.^1^, ^2^, ^3^ Other studies suggest that the presence of chronic lymphocytic thyroiditis (CLT) is associated with a lower risk of persistence/recurrence of PTC.^4^ Although the association of these findings (elevated TgAb and CLT) is common, many patients with elevated TgAb do not have CLT.^1^, ^2^, ^5^, ^6^, ^7^ Thus, it is possible that in patients with elevated TgAb, the risk of tumor persistence/recurrence differs between those with and without associated CLT, with a lower risk being expected for the former.^4^ In fact, some studies have demonstrated this protective effect of CLT specifically in patients with elevated TgAb.^5^, ^7^ However, other series found no influence of CLT on the evolution of these patients.^1^, ^2^ In contrast, one study demonstrated a higher risk of persistent/recurrent disease in patients with PTC and elevated TgAb when the latter were associated with CLT (compared to nonspecific TgAb and not related to CLT).^6^

This divergence in the results, together with the limitations of the studies that were retrospective and included a limited number of patients with elevated TgAb,^1^, ^2^, ^5^, ^6^, ^7^ shows that the influence of CLT on tumor persistence/recurrence in patients with TgAb remains undefined. In addition, tumor persistence and recurrence are distinct outcomes and their separate analysis is desirable.^3^

In view of the need for further, ideally prospective, studies that include a larger number of patients and are specifically designed,^6^, ^7^ we conducted this prospective study to evaluate the influence of CLT on the risk of persistence and recurrence of PTC in patients with negative Tg but elevated TgAb after initial therapy.

## Methods

This was a prospective study. The study was approved by the Ethics Committee on Research of our Institution (n° 411.326) and informed consent was obtained from each patient.

Patients consecutively seen at our institution who met the following criteria were selected: (i) diagnosis of PTC; (ii) submitted to total thyroidectomy followed by ablation with ^131^I (1.1–5.5 GBq); (iii) apparently complete tumor Resection and post-therapy Whole-Body Scanning (RxWBS) showing no ectopic uptake; and (iv) clinical examination showing no anomalies, basal Tg < 1 ng/mL, and elevated TgAb 8–12 months after ablation.^8^ Patients with microcarcinoma restricted to the thyroid or with the noninvasive encapsulated follicular variant of PTC were not included. Ablation was also recommended for patients with 1–4 cm PTC confined to the thyroid (*n* = 36) but who had other features (age ≤ 18 years or >45 years, multicentric tumor, elevated Tg or TgAb after the thyroidectomy). The patients were divided into two groups: Group A, with CLT on histology; Group B, without CLT on histology.

Neck ultrasonography (US), stimulated Tg, and diagnostic WBS (DxWBS) were obtained from all patients during initial assessment.^8^ Patients with a stimulated Tg > 1 ng/mL without disease on DxWBS and US were evaluated by chest computed tomography (CT), technetium-99m-methoxyisobutylisonitrile (^99m^Tc-MIBI) scintigraphy, and fluorodeoxyglucose positron emission tomography (FDG-PET)/CT.^8^ In the case of patients without disease in this first assessment, Tg, TgAb and US were obtained at intervals of 6 months. In addition, chest CT was performed annually in patients with tumors >4 cm, extrathyroid extension or lymph node metastases, and every 2 years in patients with tumors ≤4 cm restricted to the thyroid, while Tg remained negative and TgAb continued to be positive. If TgAb elevation or positive Tg was observed at any time during follow-up, chest CT, ^99m^Tc-MIBI scans and FDG-PET/CT were performed. TSH was maintained ≤0.5 mIU/L.

Eight to 12 months after ablation with ^131^I, serum Tg was measured by a radioimmunometric assay (ELSAhTG, CIS Bio International), with a functional sensitivity of 1 ng/mL. TgAb were determined by a chemiluminescent assay (Immulite, Diagnostic Products Corp.), with a reference value of up to 40 IU/mL.

CLT was defined when diffuse lymphocyte infiltration was present in the area of normal thyroid tissue.^2^

Means were compared between groups by Student *t*-test or the nonparametric Mann–Whitney *U* test. Fisher's exact test or *χ*^2^ test was used to detect differences in the proportion of cases. A *p*-value <0.05 was considered significant.

## Results

Groups A and B were similar in terms of sex, age, lymph node metastases, TNM stage and risk category,^9^ and TgAb concentrations after ablation (Table 1). The time of follow-up ranged from 60 to 140 months (median 96 months).

**Table 1:** Characteristics of the patients of Groups A and B.

	Group A (*n* = 45)	Group B (*n* = 68)
*Sex*		
Women	42 (93.3%)	61 (89.7%)
Men	3 (6.6%)	7 (10.2%)

*Age [range (median), years]*	13–72 (46)	18–74 (48)
*Risk classification*^9^		
Low risk	18 (40%)	26 (38.2%)
Intermediate risk	27 (60%)	42 (61.7%)

*Lymph node metastases*	20 (44.4%)	28 (41.2%)
*Stage*^9^		
I	24 (53.3%)	40 (58.8%)
II	4 (8.9%)	6 (8.8%)
III	9 (20%)	14 (20.6%)
IVA	8 (17.7%)	8 (11.7%)

*TgAb [range (median), IU/mL]*^a^	65–2845 (556)	76–2567 (568)
*Time of follow-up [range (median), months]*	60–140 (96)	62–140 (96)

Group A, with chronic lymphocytic thyroiditis (CLT) on histology; Group B, without CLT on histology.

TgAb, antithyroglobulin antibodies.

a 8–12 months after initial therapy.

8–12 months after ablation, persistent disease was detected in 3 patients of Group A (6.6%) and in 6 of Group B (8.8%) (*p* = 1.0), including lymph node metastases in 2 patients of Group A (4.4%) and in 4 of Group B (5.9%) (*p* = 1.0) and distant metastases in 1 patient of Group A (2.2%) and in 2 of Group B (2.9%) (*p* = 1.0). Neck US showed lymph node metastases in four patients. DxWBS revealed ectopic uptake in two patients and CT showed lymph nodes in the topography of ectopic uptake. In another patient, DxWBS showed pulmonary metastases, but a chest CT was normal. DxWBS was negative in two patients, but pulmonary metastases were detected by chest CT in one and bone metastases were detected by FDG-PET/CT in the other.

During follow-up of the 104 patients without persistent disease, recurrences were diagnosed in 2 patients of Group A (4.7%) and in 5 of Group B (8%) (*p* = 0.7), including lymph node metastases in 2 patients of Group A (4.7%) and in 4 of Group B (6.4%) (*p* = 1.0) and distant metastases in any of the patients of Group A and in 1 patient of Group B (1.6%) (*p* = 1.0). US showed lymph node metastases in 5 patients, CT revealed pulmonary metastases in one, and FDG-PET/CT was positive in another patient.

Considering both persistent and recurrent disease after initial therapy, structural disease was detected in 5 patients of Group A (11.1%) and in 11 of Group B (16.1%) (*p* = 0.58), including lymph node metastases in 4 patients of Group A (8.8%) and in 8 of Group B (11.7%) (*p* = 0.76) and distant metastases in 1 patient of Group A (2.2%) and in 3 of Group B (4.4%) (*p* = 1.0).

We separately analyzed the outcomes for low-risk and intermediate-risk patients classified according to ATA.^9^ In intermediate-risk patients, persistent disease was diagnosed in 3 patients of Group A (11.1%) and in 6 of Group B (14.3%) (*p* = 1.0) and recurrences occurred in 2 patients of Group A (8.3%) and 3 of Group B (8.3%) (*p* = 1.0), totaling 5 patients in Group A (11.1%) and 9 in Group B (13.2%) (*p* = 1.0). In the low-risk group, 9 of the patients had persistent disease and recurrences were diagnosed in any of the patients of Group A and in 2 of Group B (7.7%) (*p* = 0.51).

There was no case of death related to the disease.

## Discussion

First, it should be highlighted that, in contrast to previous studies,^1^, ^2^, ^5^, ^6^, ^7^ this was a prospective study. To the best of our knowledge, this is the largest study evaluating the influence of CLT specifically in patients with elevated TgAb. The minimum time of follow-up was 5 years and it is known that 80% of recurrences occur in these first years.^2^ Since they are distinct outcomes, persistent and recurrent disease were analyzed separately and combined.

It is known that many patients with PTC with persistently elevated TgAb after initial therapy do not have CLT; 60% in the present series and 30%,^7^ 60%^2^, ^5^ and 65%^1^, ^6^ in previous studies. It can be assumed that the persistence of elevated TgAb after treatment of PTC in the absence of underlying CLT will indicate residual disease. In addition, CLT has been associated with better evolution of PTC.^4^ This supports the hypothesis that CLT is associated with a lower risk of persistent/recurrent disease in patients with elevated TgAb after treatment of PTC.

Evaluating specifically patients with elevated TgAb after initial therapy, this protective effect of CLT was demonstrated in two series,^5^, ^7^ but was not confirmed by other authors.^1^, ^2^, ^6^ Our results also showed no influence of CLT on the rate of persistent or recurrent disease in patients with elevated TgAb. Moreover, one study showed worse evolution of patients with elevated TgAb when associated with CLT.^6^ In view of the divergent results,^1^, ^2^, ^5^, ^6^, ^7^ present study, it is possible that differences in TgAb epitopes^6^ and in the cell subpopulation of the lymphocyte infiltrate^10^ explain the heterogeneity of the influence of CLT on the evolution of PTC. As in the present study, the lack of influence of CLT on recurrent disease has recently been reported for patients without TgAb.^11^

Finally, although previous studies found no difference in the rate of persistent/recurrent disease, they report lower tumor aggressiveness on initial presentation in patients with CLT.^12^, ^13^, ^14^ Although it was not the objective of the present study, we found no difference in the presence of lymph node metastases, risk category or tumor stage between patients with versus without CLT.

## Conclusion

In conclusion, our results do not support the hypothesis that CLT on histology is associated with a lower risk of persistent or recurrent disease, at least in patients with persistently elevated TgAb after initial therapy of PTC.

## Funding

This research did not receive any specific grant from any funding agency in the public, commercial or not-for-profit sector.

## Conflicts of interest

The authors declare no conflicts of interest.
